# Expression and function of Smad7 in autoimmune and inflammatory diseases

**DOI:** 10.1007/s00109-021-02083-1

**Published:** 2021-05-31

**Authors:** Yiping Hu, Juan He, Lianhua He, Bihua Xu, Qingwen Wang

**Affiliations:** 1 Department of Rheumatism and Immunology, Peking University Shenzhen Hospital, Shenzhen, 518036 Guangdong China; 2 Shenzhen Key Laboratory of Inflammatory and Immunology Diseases, Shenzhen, 518036 Guangdong China

**Keywords:** Transforming growth factor-β, Smad7, Regulation

## Abstract

Transforming growth factor-β (TGF-β) plays a critical role in the pathological processes of various diseases. However, the signaling mechanism of TGF-β in the pathological response remains largely unclear. In this review, we discuss advances in research of Smad7, a member of the I-Smads family and a negative regulator of TGF-β signaling, and mainly review the expression and its function in diseases. Smad7 inhibits the activation of the NF-κB and TGF-β signaling pathways and plays a pivotal role in the prevention and treatment of various diseases. Specifically, Smad7 can not only attenuate growth inhibition, fibrosis, apoptosis, inflammation, and inflammatory T cell differentiation, but also promotes epithelial cells migration or disease development. In this review, we aim to summarize the various biological functions of Smad7 in autoimmune diseases, inflammatory diseases, cancers, and kidney diseases, focusing on the molecular mechanisms of the transcriptional and posttranscriptional regulation of Smad7.

## Introduction

**Fig. 1:**
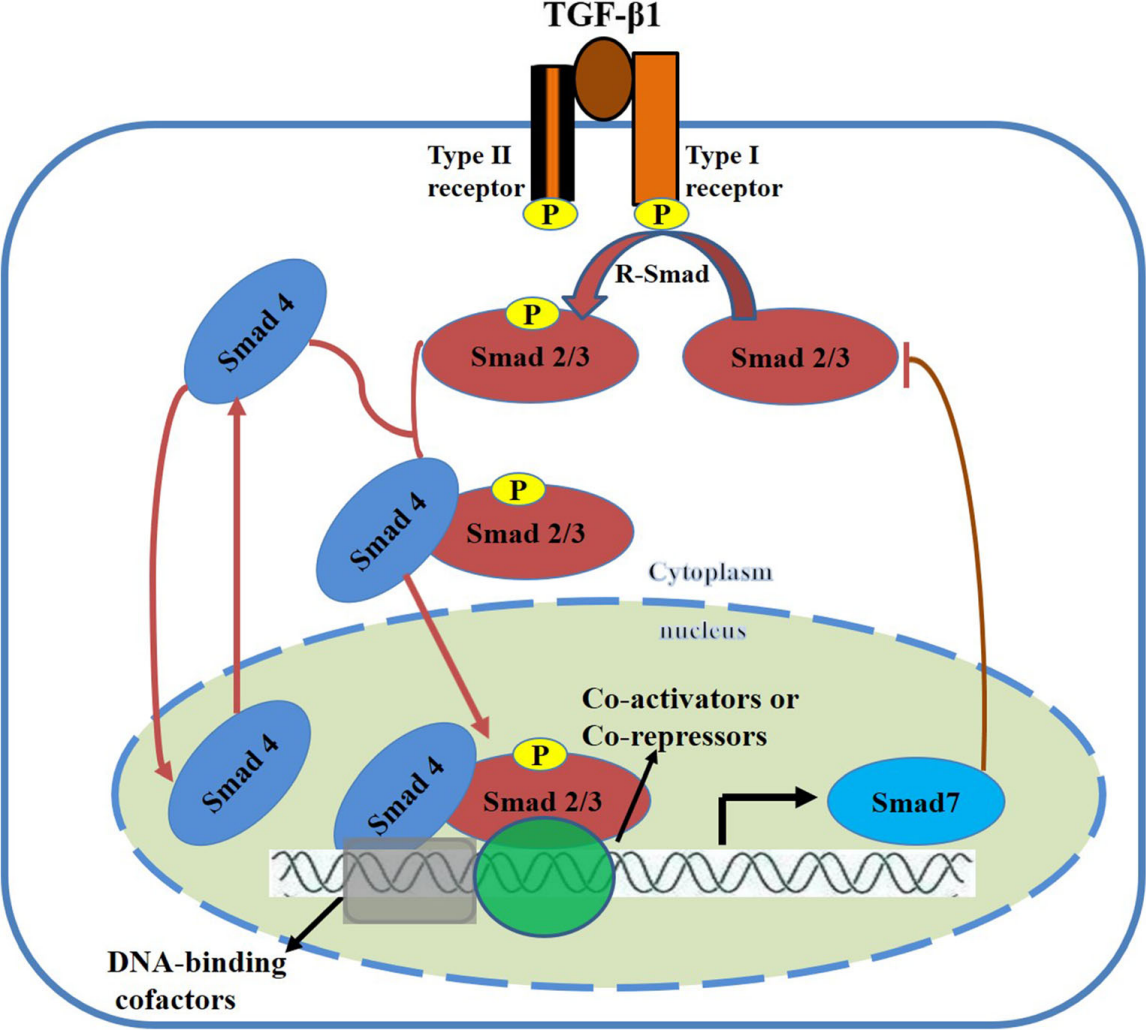
Functional and structural features of the TGF-β/Smads pathway and the role of Smad7. TGF-β1 binding to its receptor II activates the TGF-β receptor type I kinase. TGF-β receptor type I then phosphorylates Smad2 and Smad3 (R-Smad). Smad2 and Smad3 are the critical mediators of TGF-β/Smads signaling in multiple diseases. Activated Smad2/3 combines with Smad4 to form a complex that is subsequently translocated into the nucleus. The resulting Smad complex incorporates different DNA-binding cofactors that confer target gene selectivity and influence the recruitment of either transcriptional coactivators or corepressors. Several hundred genes are regulated by TGF-β, including Smad7. Smad7 plays a negative regulatory role in the TGF-β/Smads signaling pathway by either inhibiting the activation of Smad2/3 activation or blocking the nuclear translocation of Smad2/3

The *SMAD7* gene, also known as *mothers against decapentaplegic homolog 7* (*MADH7*), is located on chromosome 18 in both humans (i.e., 18q21.1) and mice (i.e., 18 51.06 cM) and encodes a protein with 426 amino acid residues [86]. Smad7 is a nuclear protein and a major negative regulator of the TGF-β signaling pathway. When TGF-β binds to the TGF-β receptor and activates the downstream signaling pathway, Smad7 is released from the nucleus into the cytoplasm, where it either inhibits the phosphorylation of Smad2/3 or induces the degradation of TGF-β receptor I and Smad2/3 [10, 50] (Fig. 1). A reduction in the phosphorylation of Smad2/3 disrupts its heterodimerization with Smad4, a common partner [5]. Smad7 is a member of the I-Smads (inhibitory Smads) family, alongside Smad6, and plays a key role in regulating signal transduction by the TGF-β family cytokines (Fig. 1). Smad6 and Smad7 are inhibitory Smads that serve as decoys that interfere with Smad–receptor and Smad–Smad interactions [63]. In contrast to R-Smads (receptor-regulated Smads) or Co-Smad (common Smad), Smad7 lacks an N-terminal MH1 domain and a phosphorylation site with type I receptors at the C-terminal tail despite having a conserved C-terminal MH2 domain [65, 99]. Moreover, Smad7 is able to bind to the DNA elements containing the minimal Smad-binding element (SBE) (CAGA) box and affects the formation of TGF-β signaling-induced functional Smad-DNA complexes [52].

Smad7 overexpression is known to inhibit the DNA binding activity of NF-κB, the translocation of NF-κB to the nucleus, the transcriptional activity of NF-κB/p65, and NF-κB-dependent inflammatory responses, suggesting a close functional relationship between Smad7 and NF-κB [69, 95]. In fact, Smad7 can induce the expression of the nuclear factor of kappa light polypeptide gene enhancer in B cell inhibitor alpha (IκBα), an NF-κB inhibitor. TGF-β inhibits NF-κB activation in an IκBα-dependent manner by inducing Smad7 [9, 51]. Inhibition of the NF-κB signaling pathway activity may be the key mechanism by which TGF-β/Smad7 is involved in inflammatory diseases [72].

TGF-β and Wnt signals undergo complex exchanges, in which TGF-β is able to both promote and suppress Wnt signaling [25, 39]. Smad7 is required for the activation of glycogen synthase kinase 3β (GSK3β) and for the stabilization of β-catenin by TGF-β. Moreover, Smad7 can associates with β-catenin and mediates the TGF-β-induced apoptosis in PC-3U human prostate cancer and HaCaT cells [19]. β-catenin is also a key component of adherens junctions. In this context, Smad7 has been shown to inhibit the activation of β-catenin and increase the expression of β-catenin and E-cadherin in breast cancer and hepatocellular carcinoma, respectively [98, 108]. In conclusion, Smad7 has different regulatory functions in multiple diseases.

TGF-β and the bone morphogenetic proteins (BMPs) transcriptionally induce Smad7; then Smad7 can negatively regulate the TGF-β and BMP signaling pathways [99]. Overexpression of Smad7 in BMP-sensitive lymphoma cells results in their conversion into BMP-resistant cells. Therefore, the upregulation of Smad7 is necessary to prevent cancer cells from adversely affecting BMPs [32]. TGF-β has been shown to activate MAPKs, including ERK, JNK, and P38, in a cell-specific manner, with Smad7 possibly playing an important role in this process. Additionally, Smad7 can independently activate JNK signaling and is essential for JNK-mediated apoptosis [64]. In prechondrogenic cells, downregulation of the BMP-activated p38 MAPK pathway may be the mechanism underlying Smad7-mediated inhibition of chondrocyte differentiation [33]. These results indicate that Smad7 not only acts as an antagonist of the TGF-β/BMP signaling pathway but also has cross-regulatory effects on other signaling pathways. In this article, we review the factors that regulate Smad7 expression and summarize its biological role in multiple diseases.

## Transcriptional regulation of Smad7

**Fig. 2:**
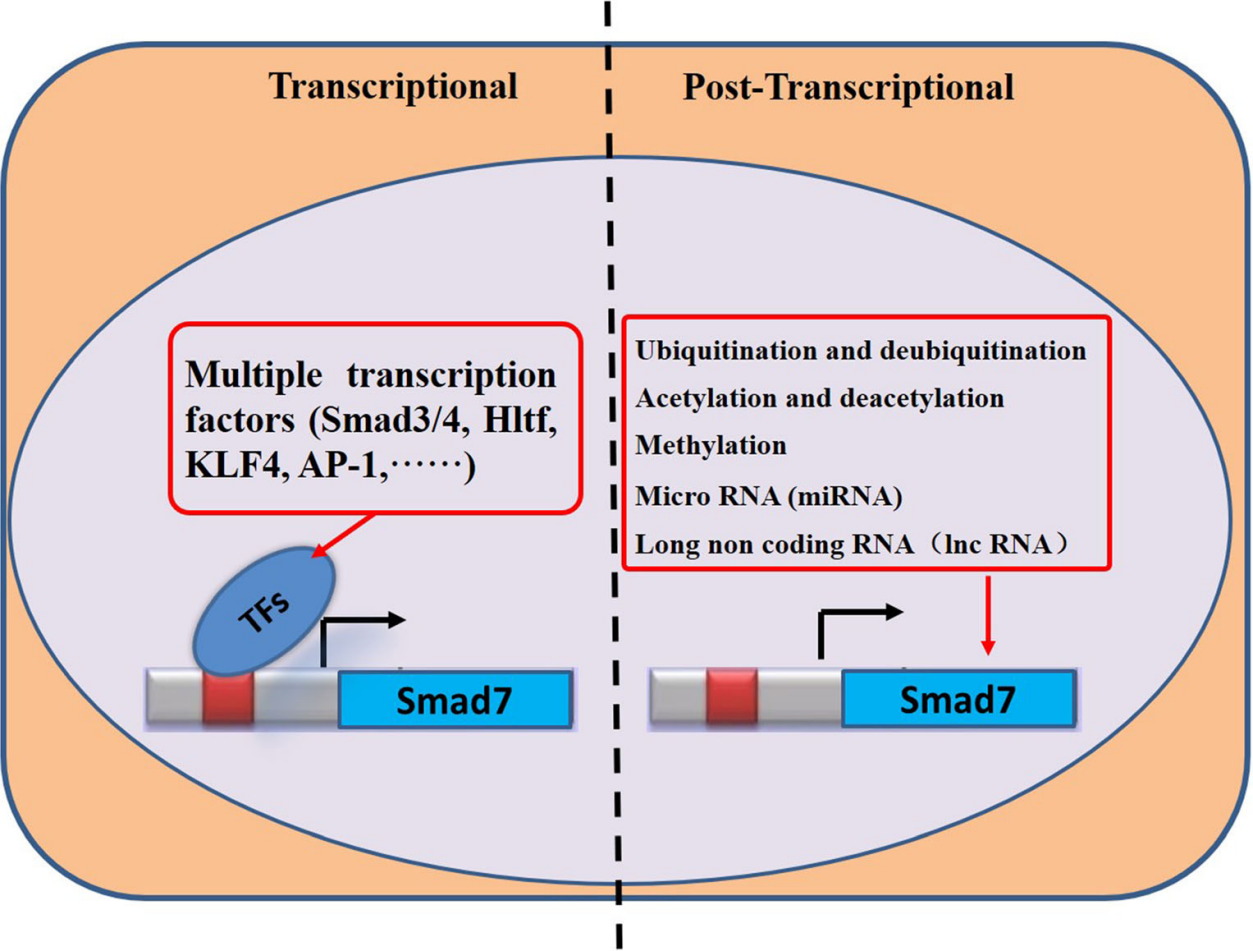
Summary of factors regulating the expression of Smad7. Smad7 can be regulated at both the transcriptional and posttranscriptional levels. A variety of transcription factors (TFs) regulate the expression of Smad7 at the transcriptional level. The regulation factor of Smad7 post-transcription involves ubiquitination, deubiquitylation, acetylation, deacetylation, methylation, miRNAs, and lncRNAs

A number of factors regulate the expression and function of Smad7 (Fig. 2). When von Gersdorff et al. [94] deleted Smad2, Smad3, and Smad4 in different cells, they found that TGF-β induced the transcription of the human *SMAD7* gene by promoting binding of the Smad3 and Smad4 transcription factors to the *SMAD7* proximal promoters. However, Smad2 was not necessary for activating *SMAD7.* Byung-Chul Kim [41] reported that Jun activation domain-binding protein 1 (Jab1)/CSN5, a constituent of the COP9 signalosome complex, stimulates the translocation of Smad7 from the nucleus to the cytoplasm and promotes the degradation of Smad7 to enhance the TGF-β-induced transcriptional activity. Denissova et al. [18] found that Ski inhibits the basal activity of the *SMAD7* promoter in a Smad-binding element (SBE)-dependent manner; mutations in the SBE abolished this inhibition. As a bona fide transcription factor, helicase-like transcription factor (Hltf) plays an important role in DNA damage repair and increases brain-cell apoptosis. It has been reported that Hltf reduces the availability of the *SMAD7* transcript in the brain tissues [30]. Furthermore, TGF-β signaling is upregulated in hepatocellular carcinoma (HCC). Sun et al. [88] demonstrated that the Krüppel-like factor 4 (KLF4) protein physically interacts with the *SMAD7* promoter. Loss of KLF4 expression in primary HCC was closely correlated with decreased *SMAD7* expression and an exacerbated TGF-β signaling pathway during oncogenesis. On the other hand, the overexpression of KLF4 suppressed TGF-β signaling during oncogenesis by activating the transcription of *SMAD7*. A decrease in the expression of YAP/TAZ or inhibition of its nuclear translocation can enhance the activity of the transcriptional factor AP-1, upregulating Smad7 expression. Thus, YAP/TAZ synergizes with the transcription factors AP-1 and Smad7 to regulate TGF-β signaling in human dermal fibroblasts [76]. Upon TGF-β stimulation, Evi-1 and its corepressor CtBP are recruited to the *SMAD7* promoter, where they inhibit the transcription and expression of *SMAD7*. This recruitment of Evi-1 in the promoter region reduces TGF-β-induced histone acetylation, which is coincident with the repression of *SMAD7* gene expression [3].

The *SMAD7* promoter is regulated by NF-κB, a transcription factor that plays an important role in inflammation and immunity. Expression of the p65 subunit of NF-κB has been shown to inhibit the activity of the *SMAD7* promoter; moreover, co-expression of the NF-κB inhibitor IκB can further suppress the activity of the *SMAD7* promoter [69]. The transcriptional co-regulator p300 is an essential component of Smad-dependent and TGF-β-induced biological functions. NF-κB, in cooperation with p300, downregulates the activity of the germ-line (GL) γ2b promoter activity by increasing the gene expression of Smad7 in B cells [82]. Treatment with p300 siRNA reduced Smad7 mRNA and TGF-β activity in neonatal cardiac fibroblasts [13]. IFN-γ has been shown to activate signal transducer and activator of transcription-1 (STAT-1) homodimerizes, translocating them to the nucleus to increase the transcription of *SMAD7* in rat mammary epithelial cells [85]. STAT-1 regulates Smad7 expression in serous papillary endometrial cancer [40]. The muscle regulatory factor (MyoD) binds to and transactivates the proximal promoter region of *SMAD7*. Interestingly, Smad7 also directly interacts with MyoD to enhance the transcriptional activity of MyoD, creating a positive feedback loop for inducing Smad7 expression and promoting MyoD-driven myogenesis in skeletal muscle cells [21, 47].

## Posttranscriptional regulation of Smad7

The regulatory factors involved in the posttranscriptional regulation of Smad7 include ubiquitination and deubiquitination, acetylation and deacetylation, methylation, microRNAs (miRNAs), and long noncoding RNAs (lncRNAs) (Fig. 2).

### Ubiquitination and deubiquitination

Smad proteins are sumoylated. However, the functional implications of this posttranslational modification remain unresolved. The Smad-induced expression of SMAD7 provides negative feedback by recruiting SMURF (Smad ubiquitination-related factor) to the TGF-β and BMP receptors for polyubiquitylation and degradative endocytosis [62]. Smad ubiquitination regulatory factor 2 (Smurf2), a ubiquitin ligase for Smad, plays a key regulatory role in the TGF-β signaling pathway during renal fibrosis, which is mainly dependent on the degradation of ubiquitinated Smad7 and Smad2 [74]. Many ubiquitin-protein ligases (E3s) target both themselves and their substrates for degradation [97]. Smurf2 is a part of this negative feedback loop and binds to Smad7 to induce the export and enrichment of activated TGF-β receptors, causing degradation of TGF-β receptors and Smad7 via the proteasomal and lysosomal pathways [38, 83].

Itch is an E3 ubiquitin ligase that positively regulates TGF-β signaling and the subsequent epithelial-to-mesenchymal transition (EMT)-related gene expression. Itch is an important regulator of Smad7 activity that mediates the ubiquitination and subsequent degradation of Smad7 [75]. Arkadia is an E3 ubiquitin ligase that is required for TGF-β signaling during EMT cell transition. It stimulates the transition of renal tubular epithelial cells to mesenchymal cells by inducing degradation of SMAD7 protein (invariant mRNA levels) [58]. Arkadia is widely expressed in mammalian tissues and interacts physiologically with Smad7, inducing its polyubiquitination and degradation, thereby enhancing the TGF-β signaling pathway [46]. Aragon et al. [6] found that WW-WW pairs in Smad regulators form functional units that have evolved to recognize PY-containing regions of variable lengths and complexity, including composite PY/phospho-Ser/Thr motifs in R-Smads and simple PY motifs in Smad7. These features expand the functional versatility of E3 ubiquitin ligases by facilitating need-based optimization of the interacting surfaces. Smad7 and Smurf1/2 act as partners for targeting TGF-β receptors for ubiquitination. Axin is a scaffold protein involved in TGF-β signaling that cooperates with Arkadia to promote Smad7 ubiquitination and enhances the degradation of Smad7. Axin significantly decreases the half-life of Smad7 and induces its nuclear export [57]. RNF12 specifically binds to Smad7 and induces its polyubiquitination and degradation [102].

The deubiquitinating enzyme USP26 is a newly discovered member of the TGF-β negative feedback loop. TGF-β rapidly increases the expression of USP26 and stabilizes Smad7 by mediating its deubiquitination. Rapid degradation of Smad7 after the knockdown of USP26 resulted in the stable expression of TGF-β receptors and elevated levels of p-Smad2 [43]. USP11 is a deubiquitinating enzyme that interacts with the inhibitory Smads that enhance TGF-β signaling. Moreover, USP11 interacts with deubiquitinates, the type I TGF-β receptor (ALK5), and enhances TGF-β-induced gene transcription [4]. OUT domain-containing protein 1 (OTUD1) was found to inhibit breast cancer stem-cell traits and metastasis via deubiquitination of Smad7 [103]. CYLD lysine-63 deubiquitinase, implicated in inherited cylindromatosis, is an enzyme that regulates TGF-β signaling in T cells and modulates the development of Tregs via deubiquitination of Smad7 [104].

### Acetylation and deacetylation

Histone deacetylases (HDACs) determine the acetylation levels of core histones and modulate gene expression. HDAC2 plays a crucial role in the activation of hepatic stellate cells (HSCs), possibly by suppressing the expression of Smad7, which is a negative modulator of HSC activation and liver fibrosis [55]. Simonsson et al. [84] demonstrated that the interaction between specific histone deacetylases (HDACs) and Smad7 is dependent on the C-terminal MH2 domain of Smad7. These HDACs can mediate Smad7 deacetylation, and HDAC1-mediated deacetylation of Smad7 decreases the stability of Smad7 by enhancing its ubiquitination. SIRT1, a class III histone deacetylase, directly interacts with the N-terminus of Smad7 and promotes the deacetylation of Smad7. SIRT1 can accelerate Smurf1-mediated ubiquitination and Smad7 degradation, resulting in Smad7 instability. SIRT1 inhibits Smad7- and TGF-β-induced mesangial cell apoptosis by accelerating the degradation of Smad7 and inhibiting the activation of caspase-3 and poly (ADP-ribose) polymerase (PARP) [49]. Prothymosin α (ProT), a highly conserved acidic nuclear protein, binds to Smad7 and enhances its acetylation by displacing HDAC1 from Smad7. ProT enhances Smad7 acetylation to stabilize Smad7 and inhibit TGF-β signaling, thereby contributing to the pathogenesis of emphysema [87]. In addition to the review cited earlier, there are also studies proving that competition between the ubiquitination and acetylation of overlapping lysine residues constitutes a novel mechanism to regulate Smad7 and related protein expression [24].

### Methylation

The enhancer of zeste homolog 2 (EZH2) is a methyltransferase that induces histone H3 lysine 27 trimethylation (H3K27me3). It plays an important role in mediating renal fibrosis by enhancing Smad7 degradation, Smad3 phosphorylation, and TGF-β receptor 1 expression [105]. Jumonji AT-rich interactive domain 1 B (JARIDIB) is a histone demethylase and a member of the JmjC domain-containing (JMJD) family. It synergizes with TGF-β-inducible early gene-1 (TIEG1) to repress the *SMAD7* promoter. JARID1B and TIEG1 inhibit *SMAD7* transcriptional activity and antagonize skin cancer development [42]. SET domain bifurcated 1 (SETDB1) is a histone methyltransferase that regulates the expression of Smad7 in breast cancer (BRC) cells. Gene network analysis and cell experiments revealed that SETDB1 knockdown promoted the upregulation of *SMAD7* in BRC. SETDB1 regulates the methylation of the H3K9 histone in the *SMAD7* promoter region, resulting in altered *SMAD7* expression, which may be a major mechanism. However, the mechanism by which SETDB1 directly regulates *SMAD7* expression remains to be elucidated and requires further study [79].

### MicroRNA

miRNA-21 can inhibit the expression of Smad7 and promote the differentiation of T helper 17 (Th17) cells, thereby mediating the development of experimental autoimmune encephalomyelitis [68]. miRNA-21-5p is highly expressed in the non-small cell lung cancer (NSCLC) cells, in which it promotes disease progression by directly targeting and promoting the *SMAD7* expression. Carboplatin suppresses NSCLC invasiveness by suppressing miRNA-21 expression and upregulating *SMAD7* expression [53, 56]. miRNA-367 promotes the invasiveness and metastasis of human pancreatic cancer cells by directly targeting the 3-untranslated region (3-UTR) of *SMAD7*, downregulating its expression of Smad7, and enhancing the TGF-β/Smad signaling pathway[107]. Similarly, miRNA-590-5p can directly target the 3′UTR of *SMAD7* and reduce its expression, thereby indirectly protecting and stabilizing Runt-related transcription factor 2 protein and promoting osteoblast differentiation [93]. miRNA-182 potentiates TGF-β-induced EMT and cancer cell metastasis by inhibiting *SMAD7* expression [101]. miRNA-497 acts as a direct negative regulator of *SMAD7* expression, inhibiting breast cancer cell growth and invasiveness [59].

### Long noncoding RNA

Feng et al. [20] reported that Erbb4-IR is a novel lncRNA that plays a key role in TGF-β/Smad3 signaling-mediated renal fibrosis. A binding site for Erbb4-IR was found on the *SMAD7* gene 3-UTR, which targeted and suppressed *SMAD7* reporter activity. Erbb4-IR correlates negatively with *SMAD7* expression and either inhibits or promotes TGF-β/Smad3-mediated renal fibrosis in vivo and in vitro. The lncRNA psoriasis susceptibility-related RNA gene induced by stress (PRINS) is significantly upregulated in patients with diabetes compared to that in healthy controls and is associated with the development of kidney disease. In addition, since the expression of PRINS correlates positively with *SMAD7* expression, it is likely that PRINS exerts its biological function by upregulating *SMAD7* expression [35].

## Smad7 in autoimmune disease

The TGF-β signaling pathway is involved in multiple biological processes. As a member of the I-Smad family, Smad7 regulates TGF-β signaling either in the cytoplasm or in the nucleus. Smad7 is also involved in multiple biological processes, including cell proliferation, differentiation, apoptosis, adhesion, and migration. The role of Smad7 in immune diseases has been studied extensively in the past few years due to the scientific advancements in the field.

### Experimental autoimmune encephalomyelitis (EAE)

Kleiter et al. [44] found that the SMAD7 protein is overexpressed in the spinal cords of the SJL/J mouse and DA rat models of EAE. The in vivo abrogation of Smad7 expression using specific antisense oligonucleotides (ODN) inhibits EAE development, as TGF-β signaling is enhanced in the peripheral immune system, and presumably in the central nervous system (CNS). Nanduri et al. [70] confirmed that Smad7 is overexpressed in the CD4+ T cells in a murine model of active EAE. In vitro, studies in the mouse EL-4 cell line research showed the 1,25(OH)_2_D_3_-mediated repression of *SMAD7* via formation of a vitamin D receptor (VDR)-Smad3-HDAC2 regulatory complex on its promoter, which contributes to the differentiation of an inflammatory T helper cell subset in the CNS. Smad7 plays a key role in the pathogenesis process of EAE. Lukas et al. [60] found that deleting Smad7 specifically in dendritic cells (DCs) prevented the development of EAE in a murine model. The primary pathological outcome of Smad7 deletion is an increase in the number of protective regulatory T cells (Tregs) and a reduction in the number of encephalitogenic effector T cells in the CNS.

Kleiter et al. [45] confirmed that Smad7 is overexpressed in peripheral-blood CD4+ T cells of patients with multiple sclerosis during a relapse but not remission; furthermore, this overexpression was strongly associated with T-bet, a T helper 1 (Th1) response transcription factor. They also found that transgenic mice overexpressing Smad7 had more severe EAE pathology, with enhanced infiltration of inflammatory cells and Th1 responses in the CNS. In contrast, silencing of Smad7-specific T cells can inhibit disease and inflammation in the CNS of mice. De Andres et al. [17] first performed an in vivo transcriptome analysis of CD4+ T lymphocytes after treatment with intravenous methylprednisolone (IVMP) in patients with multiple sclerosis. Microarray analysis revealed that the change in Smad7 expression following treatment with IVMP, in relapsing–remitting in patients with multiple sclerosis (in vivo), was significantly lower than that in CD4+ T cells from healthy donors (in vitro). Similarly, Abarca-Zabalia et al. [1] confirmed that Smad7 expression was decreased in the peripheral blood CD4+ T lymphocytes of patients with remittent recurrent multiple sclerosis during both acute relapses and in remission phases. Asano et al. [8] found that the impaired Smad7-Smurf-mediated negative regulation of TGF-β signaling might contribute to the pathogenesis of scleroderma in human dermal fibroblasts.

### Rheumatic diseases

In an in vitro experiment, Chen et al. [15] found that expressing high levels of acetylated Smad7 expression in synovial cells was associated with a low activation state. They used lentiviral vector-mediated Smad7 overexpression and intra-articular injection to treat collagen-induced arthritis in mice and showed that it alleviated the pathology of rheumatoid arthritis. Smad7 knockdown in CD-1 mice increases their sensitivity to collagen-induced arthritis. Loss of Smad7 activates the TGF-β/Smad3-IL-6 and NF-κB pathways and, in turn, induces Th1/Th17 differentiation and synovial inflammation. This may be a major pathogenic mechanism of rheumatoid arthritis [106].

Ultraviolet A1 phototherapy can decrease *SMAD7* gene expression in localized scleroderma, indicating that Smad7 plays an important role in its pathology [48]. Autoimmune disorders are the main mechanisms of multiple sclerosis. In intestinal biopsies from patients with multiple sclerosis, Smad7 was found to favor the expansion of intestinal CD4+ T cells toward an inflammatory phenotype and promote the migration of intestinal CD4+ T cells to the CNS [29]. Yang et al. [100] found that expression of Smad7 was decreased in patients with systemic lupus erythematosus (SLE) patients compared to healthy controls, suggesting that Smad7 may be involved in the progression of SLE.

## Smad7 in inflammatory diseases

The mechanisms underlying the actions of inflammatory pathways are an important subject of research and provide therapeutic targets for diseases such as inflammatory bowel disease (IBD). TGF-β1 is produced by both immune and nonimmune intestinal cells and has immunomodulatory properties that play a key role in the pathogenesis of IBD. Smad7 overexpression significantly reduced the activity of TGF-β1 activity. However, inhibiting Smad7 expression using the Smad7 specific antisense oligonucleotide mongersen activated TGF-β1 [7]. Garo et al. [23] found that Smad7 mediates intestinal inflammation by limiting the PDL2/1-PD1 axis in DCs and CD4+ T cells in mice. Smad7 depletion in DCs and T cells can enhance the expression and signaling transmission of TGF-β and PDL2/1-PD1, thereby inducing Treg cell polarization and attenuating inflammation. Tang et al. [90] reported T cell infiltration occurs in the lamina propria of patients with CD. In addition, expression of the short splice form of CYLD (sCYLD) and Smad7 was reported to be higher than in controls. Transgenic expression of sCYLD and Smad7 in mice inhibits the differentiation of Treg cells and Th17 cells and increases Th1 cell differentiation in T cells. The sCYLD–Smad7 complex inhibits TGF-β in T cells and recruits Smad7 to the nucleus, thereby inhibiting Smad3/4 activity. These effects prevent normal immune regulation mechanisms and lead to colitis in mice. TGF-β1/Smad3 signaling can be restored by treatment with a specific antisense oligonucleotide that specifically knocks down *SMAD7* expression and inhibits the production of inflammatory cytokines, attenuating colitis in mice [67]. According to a recent in-depth study on Smad7, the specific inhibitor Mongersen (GED-0301; Celgene) represents a class of new drugs that exerted the expected therapeutic effect in the clinical trials of Crohn’s disease; thus, it has a potential drug for the treatment of IBD [71, 96]. Unfortunately, the study could not be completed because the Mongersen phase III clinical trial was unable to obtain satisfactory results. However, it is likely that further novel drugs targeting Smad7 will be developed in the future.

Smad7 also plays several key functions in oral mucositis; for instance, Smad7 negatively regulates both TGF-β and NF-κB signaling, thereby inhibiting inflammation and pro-inflammatory cytokines so as to reduce mucositis formation. Similarly, the overexpression of Smad7 promotes wound healing by inducing epithelial cell proliferation and inhibiting apoptosis. As a member of the Rho family of GTPases, Rac 1 is necessary for oral wound healing and keratinocyte migration. Smad7 acts as an agonist and promotes Rac 1 production and activity [10]. In oral mucositis, Smad7 can directly reduce DNA damage or promote DNA repair, using local short-term Tat-Smad7 to inhibit TGF-β-mediated growth arrest and apoptosis and TGF-β/NF-κB-mediated inflammation. However, this effect primarily relieves radiation-induced oral mucositis without affecting the process that kills radiation-induced adjacent oral cancer [61]. Smad7 is a critical mediator of TGF-β signaling that blocks proinflammatory TNF signals in MCF7 cells [31]. Smad7 plays important role in the regulation of ovarian function. It is the main mediator of TGF-β-induced apoptosis in ovarian granulosa cells in vitro. In mouse ovary cells, it acts as a negative regulator of TGF-β1 and controls TGF-β superfamily signaling, which is necessary for signal transduction in ovarian granulosa cells. Smad7 also negatively regulates growth differentiation factor 9 (GDF9) signaling in mouse ovarian cells, and GDF acts as a key oocyte-derived factor that regulates oocyte–granule cell interaction, follicular development, and ovulation [52].

## Smad7 in cancer

Smad7 modulates TGF-β signaling during tumorigenesis. While TGF-β inhibits early tumor development by inducing cell cycle arrest, apoptosis, or differentiation, it facilitates the advancement of tumors by promoting EMT, migration, invasion, and metastasis. Monteleone et al. [66] demonstrated that blocking *SMAD7* expression with antisense oligonucleotides restored TGF-β1 signaling and inhibited the expression of interferon-γ and T-bet in *Helicobacter pylori* (*H*. *pylori*)-colonized gastric biopsy specimens. In *H*. *pylori*-infected gastric mucosa, interferon-γ-induced Smad7 production prevented endogenous TGF-β1 from downregulating the ongoing tissue-damaging Th1 response. Boulay et al. [11] found that patients with colorectal cancer (CRC) with defects in Smad7 had a significantly better prognosis than did the patients with two copies of the gene. Deletion of Smad7 can enhance tumor sensitivity and inhibit TGF-β activity. Consistent with this, the amplification of Smad7 may have adverse effects. Overexpression of Smad7 in colonic adenocarcinoma (FET) cells induces tumorigenesis, principally by limiting TGF-induced growth inhibition and apoptosis. Stable expression of Smad7 in colon cancer induces liver metastasis by negatively regulating the TGF-β/Smad pathway [27]. To elaborate, analysis of human colon specimens demonstrated that Smad7 was downregulated in the CD4+ T cells in the lamina propria of patients with complicated IBD relative to those in patients with uncomplicated IBD. To assess its effect on the sensitivity of colitis-associated colorectal cancer (CAC), Smad7 was overexpressed in T cells in a transgenic mouse model. It was reported that in comparison to the nontransgenic control mice, Smad7 overexpression increased the severity of colitis and reduced the incidence of tumors. This protection was associated with the overexpression of Smad7, which in turn promoted the expression of IFN-γ and the accumulation of cytotoxic CD8+ T cells and natural killer T cells in tumors and in tissues adjacent to the tumors [77, 92]. In colon cancer transplantation experiments, the development of tumors in T cell-specific Smad7 transgenic mice was inhibited relative to that in wild-type (WT) mice, with the resistance dependent on CD4+ T cells. The expression of Smad7 in T cells led to an increase in the number of tumor-infiltrating T-bet/RORγ-t double-positive CD4+ T cells while decreasing the expression of IL-17A in CD+ T cells. The low expression of IL17A caused by the Smad7 expression in tumor-infiltrating CD4(+) T cells enabled the TNF-α-mediated killing of cancer cells both in vitro and in vivo [78].

Broderick et al. [12] identified genetic variants associated with increased CRC risk, studying correlations among the three SNPs (rs4939827, rs12953717, and rs4464148) of *SMAD7* with CRC. They reported a highly significant difference between rs4939827 and CRC. Furthermore, the three common variants of *SMAD7* (rs4464148, rs4939827, and rs12953717) were confirmed in two recent genome-wide association studies (GWAS). These variants were associated with moderate susceptibility to colorectal cancer. Another study that evaluated the association of rs4464148 with colon cancer using stratified analysis of the study showed that this association was limited to women. Preliminary evidence has also indicated that the association of rs4939827 and rs12953717 with colon cancer is found in women only [2, 91]. Blocking the TGF-β/Smad pathway while stably expressing Smad7 in colon cancer cells induced liver metastasis, indicating that the Smad signaling pathway plays an important role in inhibiting the metastasis of colon cancer [27].

The metastasis-associated 1 (MTA1) gene assists metastasis, as a part of the nucleosome remodeling and HDAC complexes, by modulating several molecular targets that are involved in transcriptional regulation. MTA1 regulates Smad7 expression by enriching the *SMAD7* promoter and is involved in the tumorigenesis and metastasis of breast cancer [80]. Similarly, OUT domain-containing protein 1 (OTUD1) inhibits breast cancer metastasis by modulating Smad7 deubiquitylation, attenuating the TGF-β-induced oncogene response [103]. The transcription of *SMAD7* is upregulated in chemically induced skin tumors and TGF-β-treated normal keratinocytes. Smad7 acts as a promoter of chemically induced skin cancers. The primary mechanism involves regulation of the survival of cells exposed to DNA damage and an increase in EGFR signaling as an upstream regulator of EGFR-mediated skin tumorigenesis [26].

Kaczorowski et al. [37] measured the expression of Smad7 in 205 patients with skin melanoma and found that expression was closely related to invasive tumor phenotypes, such as ulcerated tumors and tumors with a higher thickness and mitotic rates. However, no regional or distant metastases were observed. Thus, Smad7 can be used as a marker of aggressive tumor behavior and adverse clinical in patients with melanoma. Furthermore, stable Smad7expression and blocking of the TGF-β signaling may provide effective treatment alternatives for melanoma and bone metastasis [34]. The function of Smad7 in different types of tumors is complicated by the fact that it exerts variable effects. For example, it plays an antitumor role in melanoma and breast cancer but promotes malignant tumors, particularly those in the skin, colon, pancreas, and endometrium. Thus, in general, the type of tumor cell seems to determine whether Smad7 acts as a promoter or suppressor [89].

## Smad7 in kidney disease

The activation of Smad7 in the rat kidney tubular epithelial cell (TEC) line (NRK52E) is known to contribute to TGF-mediated renal fibrosis. Doxycycline-induced overexpression of Smad7 prevents collagen synthesis and myofibroblastic transformation by inhibiting TGF-β-induced Smad2 activation. Therefore, Smad7 plays an important role in the pathology of renal fibrosis [54]. Nine et al. [73] demonstrated that the TGF-β/Smad signaling pathway plays an important role in the regulation of peritoneal fibrosis. They used ultrasound microbubble systems to mediate the overexpression of Smad7 in a rat model of peritoneal dialysis (PD). They found that ultrasound-mediated gene transfer of Smad7 could, in fact, regulate TGF-β/Smad signaling and improve peritoneal fibrosis by inhibiting Smad2/3 activation. Although the overexpression of TGF-β plays an important role in the development of renal fibrosis, the levels of *SMAD7* protein had decreased in unilateral ureteral obstruction kidneys compared to placebo-surgery control kidneys in mouse models. The ubiquitin-dependent degradation of Smad7 plays a pathogenic role in the progression of tubulointerstitial fibrosis [22].

Wang et al. [95] found that latent TGF-β1 could prevent renal inflammation in a murine model of obstructive kidney disease. The primary mechanism involves the overexpression of Smad7, which upregulates the expression of IκBα and inhibits NF-κB activation. This result suggests that the interaction between the TGF-β and NF-κB signaling pathways plays an important role in the in vivo regulation of renal inflammation*.* Smad7 overexpression can improve renal fibrosis by increasing the expression of miRNA-29 and inhibiting the levels of miRNA-192 and miRNA-21 in obstructive nephropathy kidney cells. The primary mechanism underlying the protection that Smad7 provides to the kidneys against fibrosis is the regulation of TGF-β/Smad3 signaling, which mediates the production of these miRNAs [16]. Overexpression of Smad7 inhibited TGF-β signaling and had therapeutic effects in a mouse model of autoimmune crescentic glomerulonephritis. Similarly, the overexpression of Smad7 provides protection against diabetic renal injury and may serve as a therapeutic target for diabetic kidney complications. Thus, Smad7 plays an important protective role in the pathogenesis of autoimmune kidney diseases [14, 36].

## Smad7 in other diseases

Smad7 is expressed in T cells and functions as an intracellular inhibitor of TGF-β signaling. Infection with *Heligmosomoides polygyrus bakeri* decreases Smad7 expression in intestinal CD4+ T cells and promotes the production of the cytokines TGF-β and IL-10 in the gut. TGF-β prevents colitis by inducing Tregs [28]. The expression levels of the *SMAD7* protein in both epithelial and lamina propria cells were found to be elevated in patients with active refractory coeliac disease (RCD). This phenomenon was associated with defects in TGF-β signaling and is primarily characterized by decreased levels of p-Smad2/3 and increased Smad7 expression. Furthermore, the expression levels of IL-6 and TNF-α were reduced in the RCD mucosa biopsy sample when Smad7 expression was knocked down [81].

## Summary and conclusions

The studies reviewed earlier indicate that Smad7 acts as a negative regulator of the TGF-β pathway that modulates the occurrence and progression of many diseases. Furthermore, they indicate that depending on the disease pathogenesis, Smad7 can play different roles in disease regulation. Therefore, differences in Smad7 levels can be used as a marker in the diagnosis of multiple diseases. It also has therapeutic potential since it is known to exert immunomodulatory functions in immune diseases. Smad7 has both prophylactic and therapeutic effects on collagen-induced arthritis in mice and targets multiple pathological processes implicated in inflammatory diseases, cancer, kidney disease, and other diseases, particularly through its effects on the proliferation, apoptosis, migration, fibrosis, and inflammation in multiple cells, tissues, and organs. Smad7 participates in the differentiation of T cells and promotes activation of immune cells such as B cells and DCs by negatively regulating the TGF-β pathway and may thereby mediate the progression of immune diseases. In addition, Smad7 inhibits both NF-κB and TGF-β activation and thus plays a pivotal role in inflammatory diseases. Smad7 is known as an inhibitory Smad or a protective Smad that negatively mediates Smad3-induced fibrogenesis. Therefore, based on its mechanism of action in different diseases, Smad7 is an attractive therapeutic target. As the number of studies on Smad7 continues to increase, the role of Smad7 in disease diagnosis and treatment, as well as the molecular mechanisms underlying its action, is likely to become clearer.

Advances in our understanding of the regulation of Smad7 expression and its function in diseases have improved our understanding of the molecular mechanisms of Smad7 in multiple pathologies. Smad7 attenuates growth inhibition, fibrosis, apoptosis, inflammation, and inflammatory T cell differentiation and also promotes epithelial cells migration and disease development. Understanding which factors regulate Smad7 is useful for exploring and developing Smad7 as a therapeutic target. Given the safety considerations, developing Smad7 as a drug target may be a long-term process. However, the advancements in research methods suggest that modulation of Smad7 activity may have promising clinical applications.

## References

[1] Abarca-Zabalia J, Garcia MI, Lozano Ros A, Marin-Jimenez I, Martinez-Gines ML, Lopez-Cauce B, Martin-Barbero ML, Salvador-Martin S, Sanjurjo-Saez M, Garcia-Dominguez JM, Lopez Fernandez LA (2020) Differential expression of SMAD genes and S1PR1 on circulating CD4+ T cells in multiple sclerosis and Crohn’s disease. Int J Mol Sci 2 10.3390/ijms21020676 PMC7014376 31968593

[2] Abd El-Fattah AA, Sadik NAH, Shaker OG, Mohamed Kamal A (2018) Single nucleotide polymorphism in SMAD7 and CHI3L1 and colorectal cancer risk. Mediat Inflamm 9853192 10.1155/2018/9853192 PMC6222239 30498395

[3] Alliston T, Ko TC, Cao Y, Liang YY, Feng XH, Chang C, Derynck R (2005). Repression of bone morphogenetic protein and activin-inducible transcription by Evi-1. J Biol Chem.

[4] Al-Salihi MA, Herhaus L, Macartney T, Sapkota GP (2012). USP11 augments TGFβ signalling by deubiquitylating ALK5. Open Biol.

[5] Andrieux G, Fattet L, Le Borgne M, Rimokh R, Theret N (2012). Dynamic regulation of TGF-B signaling by TIF1γ: a computational approach. PLoS One.

[6] Aragon E, Goerner N, Xi Q, Gomes T, Gao S, Massague J, Macias MJ (2012). Structural basis for the versatile interactions of Smad7 with regulator WW domains in TGF-β pathways. Structure.

[7] Argollo M, Fiorino G, Hindryckx P, Peyrin-Biroulet L, Danese S (2017). Novel therapeutic targets for inflammatory bowel disease. J Autoimmun.

[8] Asano Y, Ihn H, Yamane K, Kubo M, Tamaki K (2004). Impaired Smad7-Smurf-mediated negative regulation of TGF-β signaling in scleroderma fibroblasts. J Clin Invest.

[9] Azuma M, Motegi K, Aota K, Yamashita T, Yoshida H, Sato M (1999). TGF-β1 inhibits NF-ĸB activity through induction of IkappaB-alpha expression in human salivary gland cells: a possible mechanism of growth suppression by TGF-β1. Exp Cell Res.

[10] Bian L, Han G, Zhao CW, Garl PJ, Wang XJ (2015). The role of Smad7 in oral mucositis. Protein Cell.

[11] Boulay JL, Mild G, Lowy A, Reuter J, Lagrange M, Terracciano L, Laffer U, Herrmann R, Rochlitz C (2003). SMAD7 is a prognostic marker in patients with colorectal cancer. Int J Cancer.

[12] Broderick P, Carvajal-Carmona L, Pittman AM, Webb E, Howarth K, Rowan A, Lubbe S, Spain S, Sullivan K, Fielding S, Jaeger E, Vijayakrishnan J, Kemp Z, Gorman M, Chandler I, Papaemmanuil E, Penegar S, Wood W, Sellick G, Qureshi M, Teixeira A, Domingo E, Barclay E, Martin L, Sieber O, Consortium C, Kerr D, Gray R, Peto J, Cazier JB, Tomlinson I, Houlston RS (2007). A genome-wide association study shows that common alleles of SMAD7 influence colorectal cancer risk. Nat Genet.

[13] Bugyei-Twum A, Advani A, Advani SL, Zhang Y, Thai K, Kelly DJ, Connelly KA (2014) High glucose induces Smad activation via the transcriptional coregulator p300 and contributes to cardiac fibrosis and hypertrophy. Cardiovasc Diabetol 89 10.1186/1475-2840-13-89 PMC4108062 24886336

[14] Chen HY, Huang XR, Wang W, Li JH, Heuchel RL, Chung AC, Lan HY (2011). The protective role of Smad7 in diabetic kidney disease: mechanism and therapeutic potential. Diabetes.

[15] Chen SY, Shiau AL, Wu CL, Wang CR (2016) Intraarticular overexpression of Smad7 ameliorates experimental arthritis. Sci Rep 35163 10.1038/srep35163 PMC5059702 27731365

[16] Chung AC, Dong Y, Yang W, Zhong X, Li R, Lan HY (2013). Smad7 suppresses renal fibrosis via altering expression of TGF-β/Smad3-regulated microRNAs. Mol Ther.

[17] De Andres C, Garcia MI, Goicoechea H, Martinez-Gines ML, Garcia-Dominguez JM, Martin ML, Romero-Delgado F, Benguria A, Sanjurjo M, Lopez-Fernandez LA (2018). Genes differentially expressed by methylprednisolone in vivo in CD4 T lymphocytes from multiple sclerosis patients: potential biomarkers. Pharm J.

[18] Denissova NG, Liu F (2004). Repression of endogenous Smad7 by Ski. J Biol Chem.

[19] Edlund S, Lee SY, Grimsby S, Zhang S, Aspenstrom P, Heldin CH, Landstrom M (2005). Interaction between Smad7 and β-catenin: importance for transforming growth factor beta-induced apoptosis. Mol Cell Biol.

[20] Feng M, Tang PM, Huang XR, Sun SF, You YK, Xiao J, Lv LL, Xu AP, Lan HY (2018). TGF-β mediates renal fibrosis via the Smad3-Erbb4-IR long noncoding RNA axis. Mol Ther.

[21] Forbes D, Jackman M, Bishop A, Thomas M, Kambadur R, Sharma M (2006). Myostatin auto-regulates its expression by feedback loop through Smad7 dependent mechanism. J Cell Physiol.

[22] Fukasawa H, Yamamoto T, Togawa A, Ohashi N, Fujigaki Y, Oda T, Uchida C, Kitagawa K, Hattori T, Suzuki S, Kitagawa M, Hishida A (2004). Down-regulation of Smad7 expression by ubiquitin-dependent degradation contributes to renal fibrosis in obstructive nephropathy in mice. Proc Natl Acad Sci U S A.

[23] Garo LP, Ajay AK, Fujiwara M, Beynon V, Kuhn C, Gabriely G, Sadhukan S, Raheja R, Rubino S, Weiner HL, Murugaiyan G (2019). Smad7 controls immunoregulatory PDL2/1-PD1 signaling in intestinal inflammation and autoimmunity. Cell Rep.

[24] Gronroos E, Hellman U, Heldin CH, Ericsson J (2002). Control of Smad7 stability by competition between acetylation and ubiquitination. Mol Cell.

[25] Guo X, Wang XF (2009). Signaling cross-talk between TGF-β/BMP and other pathways. Cell Res.

[26] Ha Thi HT, Kim HY, Lee YJ, Kim SJ, Hong S (2019). SMAD7 in keratinocytes promotes skin carcinogenesis by activating ATM-dependent DNA repair and an EGFR-mediated cell proliferation pathway. Carcinogenesis.

[27] Halder SK, Rachakonda G, Deane NG, Datta PK (2008). Smad7 induces hepatic metastasis in colorectal cancer. Br J Cancer.

[28] Hang L, Kumar S, Blum AM, Urban JF, Fantini MC, Weinstock JV (2019). Heligmosomoides polygyrus bakeri infection decreases Smad7 expression in intestinal CD4(+) T cells, which allows TGF-β to induce IL-10-producing regulatory T cells that block colitis. J Immunol.

[29] Haupeltshofer S, Leichsenring T, Berg S, Pedreiturria X, Joachim SC, Tischoff I, Otte JM, Bopp T, Fantini MC, Esser C, Willbold D, Gold R, Faissner S, Kleiter I (2019). Smad7 in intestinal CD4(+) T cells determines autoimmunity in a spontaneous model of multiple sclerosis. Proc Natl Acad Sci U S A.

[30] Helmer RA, Foreman O, Dertien JS, Panchoo M, Bhakta SM, Chilton BS (2013). Role of helicase-like transcription factor (hltf) in the G2/m transition and apoptosis in brain. PLoS One.

[31] Hong S, Lim S, Li AG, Lee C, Lee YS, Lee EK, Park SH, Wang XJ, Kim SJ (2007). Smad7 binds to the adaptors TAB2 and TAB3 to block recruitment of the kinase TAK1 to the adaptor TRAF2. Nat Immunol.

[32] Huse K, Bakkebo M, Walchli S, Oksvold MP, Hilden VI, Forfang L, Bredahl ML, Liestol K, Alizadeh AA, Smeland EB, Myklebust JH (2012). Role of Smad proteins in resistance to BMP-induced growth inhibition in B-cell lymphoma. PLoS One.

[33] Iwai T, Murai J, Yoshikawa H, Tsumaki N (2008). Smad7 inhibits chondrocyte differentiation at multiple steps during endochondral bone formation and down-regulates p38 MAPK pathways. J Biol Chem.

[34] Javelaud D, Mohammad KS, McKenna CR, Fournier P, Luciani F, Niewolna M, Andre J, Delmas V, Larue L, Guise TA, Mauviel A (2007). Stable overexpression of Smad7 in human melanoma cells impairs bone metastasis. Cancer Res.

[35] Jiao H, Xie D, Qiao Y (2019). LncRNA PRINS is involved in the development of nephropathy in patients with diabetes via interaction with Smad7. Exp Ther Med.

[36] Ka SM, Huang XR, Lan HY, Tsai PY, Yang SM, Shui HA, Chen A (2007). Smad7 gene therapy ameliorates an autoimmune crescentic glomerulonephritis in mice. J Am Soc Nephrol.

[37] Kaczorowski M, Biecek P, Donizy P, Pieniazek M, Matkowski R, Halon A (2019). SMAD7 is a novel independent predictor of survival in patients with cutaneous melanoma. Transl Res.

[38] Kavsak P, Rasmussen RK, Causing CG, Bonni S, Zhu H, Thomsen GH, Wrana JL (2000). Smad7 binds to Smurf2 to form an E3 ubiquitin ligase that targets the TGF β receptor for degradation. Mol Cell.

[39] Kawabata M, Imamura T, Miyazono K (1998). Signal transduction by bone morphogenetic proteins. Cytokine Growth Factor Rev.

[40] Kharma B, Baba T, Matsumura N, Kang HS, Hamanishi J, Murakami R, McConechy MM, Leung S, Yamaguchi K, Hosoe Y, Yoshioka Y, Murphy SK, Mandai M, Hunstman DG, Konishi I (2014). STAT1 drives tumor progression in serous papillary endometrial cancer. Cancer Res.

[41] Kim BC, Lee HJ, Park SH, Lee SR, Karpova TS, McNally JG, Felici A, Lee DK, Kim SJ (2004). Jab1/CSN5, a component of the COP9 signalosome, regulates transforming growth factor β signaling by binding to Smad7 and promoting its degradation. Mol Cell Biol.

[42] Kim J, Shin S, Subramaniam M, Bruinsma E, Kim TD, Hawse JR, Spelsberg TC, Janknecht R (2010). Histone demethylase JARID1B/KDM5B is a corepressor of TIEG1/KLF10. Biochem Biophys Res Commun.

[43] Kit Leng Lui S, Iyengar PV, Jaynes P, Isa Z, Pang B, Tan TZ, Eichhorn PJA (2017). USP26 regulates TGF-β signaling by deubiquitinating and stabilizing SMAD7. EMBO Rep.

[44] Kleiter I, Pedre X, Mueller AM, Poeschl P, Couillard-Despres S, Spruss T, Bogdahn U, Giegerich G, Steinbrecher A (2007). Inhibition of Smad7, a negative regulator of TGF-β signaling, suppresses autoimmune encephalomyelitis. J Neuroimmunol.

[45] Kleiter I, Song J, Lukas D, Hasan M, Neumann B, Croxford AL, Pedre X, Hovelmeyer N, Yogev N, Mildner A, Prinz M, Wiese E, Reifenberg K, Bittner S, Wiendl H, Steinman L, Becker C, Bogdahn U, Neurath MF, Steinbrecher A, Waisman A (2010). Smad7 in T cells drives T helper 1 responses in multiple sclerosis and experimental autoimmune encephalomyelitis. Brain Pt.

[46] Koinuma D, Shinozaki M, Komuro A, Goto K, Saitoh M, Hanyu A, Ebina M, Nukiwa T, Miyazawa K, Imamura T, Miyazono K (2003). Arkadia amplifies TGF-β superfamily signalling through degradation of Smad7. EMBO J.

[47] Kollias HD, Perry RL, Miyake T, Aziz A, McDermott JC (2006). Smad7 promotes and enhances skeletal muscle differentiation. Mol Cell Biol.

[48] Kreuter A, Hyun J, Skrygan M, Sommer A, Tomi NS, Breuckmann F, Altmeyer P, Gambichler T (2006). Ultraviolet A1 phototherapy decreases inhibitory SMAD7 gene expression in localized scleroderma. Arch Dermatol Res.

[49] Kume S, Haneda M, Kanasaki K, Sugimoto T, Araki S, Isshiki K, Isono M, Uzu T, Guarente L, Kashiwagi A, Koya D (2007). SIRT1 inhibits transforming growth factor β-induced apoptosis in glomerular mesangial cells via Smad7 deacetylation. J Biol Chem.

[50] Lan HY (2011). Diverse roles of TGF-β/Smads in renal fibrosis and inflammation. Int J Biol Sci.

[51] Lan HY, Chung AC (2012). TGF-β/Smad signaling in kidney disease. Semin Nephrol.

[52] Li Q (2015). Inhibitory SMADs: potential regulators of ovarian function. Biol Reprod.

[53] Li X, Wu X (2018). MiR-21-5p promotes the progression of non-small-cell lung cancer by regulating the expression of SMAD7. Onco Targets Ther.

[54] Li JH, Zhu HJ, Huang XR, Lai KN, Johnson RJ, Lan HY (2002). Smad7 inhibits fibrotic effect of TGF-β on renal tubular epithelial cells by blocking Smad2 activation. J Am Soc Nephrol.

[55] Li X, Wu XQ, Xu T, Li XF, Yang Y, Li WX, Huang C, Meng XM, Li J (2016). Role of histone deacetylases(HDACs) in progression and reversal of liver fibrosis. Toxicol Appl Pharmacol.

[56] Lin L, Tu HB, Wu L, Liu M, Jiang GN (2016). MicroRNA-21 regulates non-small cell lung cancer cell invasion and chemo-sensitivity through SMAD7. Cell Physiol Biochem.

[57] Liu W, Rui H, Wang J, Lin S, He Y, Chen M, Li Q, Ye Z, Zhang S, Chan SC, Chen YG, Han J, Lin SC (2006). Axin is a scaffold protein in TGF-β signaling that promotes degradation of Smad7 by Arkadia. EMBO J.

[58] Liu FY, Li XZ, Peng YM, Liu H, Liu YH (2008). Arkadia regulates TGF-β signaling during renal tubular epithelial to mesenchymal cell transition. Kidney Int.

[59] Liu J, Zhou Y, Shi Z, Hu Y, Meng T, Zhang X, Zhang S, Zhang J (2016). microRNA-497 modulates breast cancer cell proliferation, invasion, and survival by targeting SMAD7. DNA Cell Biol.

[60] Lukas D, Yogev N, Kel JM, Regen T, Mufazalov IA, Tang Y, Wanke F, Reizis B, Muller W, Kurschus FC, Prinz M, Kleiter I, Clausen BE, Waisman A (2017). TGF-β inhibitor Smad7 regulates dendritic cell-induced autoimmunity. Proc Natl Acad Sci U S A.

[61] Luo J, Bian L, Blevins MA, Wang D, Liang C, Du D, Wu F, Holwerda B, Zhao R, Raben D, Zhou H, Young CD, Wang XJ (2019). Smad7 promotes healing of radiotherapy-induced oral mucositis without compromising oral cancer therapy in a xenograft mouse model. Clin Cancer Res.

[62] Massague J (2012). TGFβ signalling in context. Nat Rev Mol Cell Biol.

[63] Massague J, Seoane J, Wotton D (2005). Smad transcription factors. Genes Dev.

[64] Mazars A, Lallemand F, Prunier C, Marais J, Ferrand N, Pessah M, Cherqui G, Atfi A (2001). Evidence for a role of the JNK cascade in Smad7-mediated apoptosis. J Biol Chem.

[65] Mochizuki T, Miyazaki H, Hara T, Furuya T, Imamura T, Watabe T, Miyazono K (2004). Roles for the MH2 domain of Smad7 in the specific inhibition of transforming growth factor-β superfamily signaling. J Biol Chem.

[66] Monteleone G, Del Vecchio BG, Palmieri G, Vavassori P, Monteleone I, Colantoni A, Battista S, Spagnoli LG, Romano M, Borrelli M, MacDonald TT, Pallone F (2004). Induction and regulation of Smad7 in the gastric mucosa of patients with Helicobacter pylori infection. Gastroenterology.

[67] Monteleone G, Caruso R, Pallone F (2012). Role of Smad7 in inflammatory bowel diseases. World J Gastroenterol.

[68] Murugaiyan G, da Cunha AP, Ajay AK, Joller N, Garo LP, Kumaradevan S, Yosef N, Vaidya VS, Weiner HL (2015). MicroRNA-21 promotes Th17 differentiation and mediates experimental autoimmune encephalomyelitis. J Clin Invest.

[69] Nagarajan RP, Chen F, Li W, Vig E, Harrington MA, Nakshatri H, Chen Y (2000) Repression of transforming-growth-factor-beta-mediated transcription by nuclear factor ĸB. Biochem J 591-6 PMC1221102 10839991

[70] Nanduri R, Mahajan S, Bhagyaraj E, Sethi K, Kalra R, Chandra V, Gupta P (2015). The active form of vitamin D Transcriptionally represses Smad7 signaling and activates extracellular signal-regulated kinase (ERK) to inhibit the differentiation of a inflammatory T helper cell subset and suppress experimental autoimmune encephalomyelitis. J Biol Chem.

[71] Neurath MF (2017). Current and emerging therapeutic targets for IBD. Nat Rev Gastroenterol Hepatol.

[72] Ng YY, Hou CC, Wang W, Huang XR, Lan HY (2005). Blockade of NFĸB activation and renal inflammation by ultrasound-mediated gene transfer of Smad7 in rat remnant kidney. Kidney Int Suppl.

[73] Nie J, Dou X, Hao W, Wang X, Peng W, Jia Z, Chen W, Li X, Luo N, Lan HY, Yu XQ (2007). Smad7 gene transfer inhibits peritoneal fibrosis. Kidney Int.

[74] Ohashi N, Yamamoto T, Uchida C, Togawa A, Fukasawa H, Fujigaki Y, Suzuki S, Kitagawa K, Hattori T, Oda T, Hayashi H, Hishida A, Kitagawa M (2005). Transcriptional induction of Smurf2 ubiquitin ligase by TGF-β. FEBS Lett.

[75] Park SH, Jung EH, Kim GY, Kim BC, Lim JH, Woo CH (2015). Itch E3 ubiquitin ligase positively regulates TGF-β signaling to EMT via Smad7 ubiquitination. Mol Cell.

[76] Qin Z, Xia W, Fisher GJ, Voorhees JJ, Quan T (2018). YAP/TAZ regulates TGF-β/Smad3 signaling by induction of Smad7 via AP-1 in human skin dermal fibroblasts. Cell Commun Signal.

[77] Rizzo A, Waldner MJ, Stolfi C, Sarra M, Fina D, Becker C, Neurath MF, Macdonald TT, Pallone F, Monteleone G, Fantini MC (2011). Smad7 expression in T cells prevents colitis-associated cancer. Cancer Res.

[78] Rizzo A, De Mare V, Rocchi C, Stolfi C, Colantoni A, Neurath MF, Macdonald TT, Pallone F, Monteleone G, Fantini MC (2014). Smad7 induces plasticity in tumor-infiltrating Th17 cells and enables TNF-alpha-mediated killing of colorectal cancer cells. Carcinogenesis.

[79] Ryu TY, Kim K, Kim SK, Oh JH, Min JK, Jung CR, Son MY, Kim DS, Cho HS (2019). SETDB1 regulates SMAD7 expression for breast cancer metastasis. BMB Rep.

[80] Salot S, Gude R (2013). MTA1-mediated transcriptional repression of SMAD7 in breast cancer cell lines. Eur J Cancer.

[81] Sedda S, De Simone V, Marafini I, Bevivino G, Izzo R, Paoluzi OA, Colantoni A, Ortenzi A, Giuffrida P, Corazza GR, Vanoli A, Di Sabatino A, Pallone F, Monteleone G (2017). High Smad7 sustains inflammatory cytokine response in refractory coeliac disease. Immunology.

[82] Seo GY, Park SR, Kim PH (2009). Analyses of TGF-β1-inducible Ig germ-line γ2b promoter activity: involvement of Smads and NF-κB. Eur J Immunol.

[83] Sim WJ, Iyengar PV, Lama D, Lui SKL, Ng HC, Haviv-Shapira L, Domany E, Kappei D, Tan TZ, Saei A, Jaynes PW, Verma CS, Kumar AP, Rouanne M, Ha HK, Radulescu C, Ten Dijke P, Eichhorn PJA, Thiery JP (2019). c-Met activation leads to the establishment of a TGFβ-receptor regulatory network in bladder cancer progression. Nat Commun.

[84] Simonsson M, Heldin CH, Ericsson J, Gronroos E (2005). The balance between acetylation and deacetylation controls Smad7 stability. J Biol Chem.

[85] Soto P, Price-Schiavi SA, Carraway KL (2003). SMAD2 and SMAD7 involvement in the post-translational regulation of Muc4 via the transforming growth factor-β and interferon-gamma pathways in rat mammary epithelial cells. J Biol Chem.

[86] Stolfi C, Marafini I, De Simone V, Pallone F, Monteleone G (2013). The dual role of Smad7 in the control of cancer growth and metastasis. Int J Mol Sci.

[87] Su BH, Tseng YL, Shieh GS, Chen YC, Wu P, Shiau AL, Wu CL (2016). Over-expression of prothymosin-alpha antagonizes TGFβ signalling to promote the development of emphysema. J Pathol.

[88] Sun H, Peng Z, Tang H, Xie D, Jia Z, Zhong L, Zhao S, Ma Z, Gao Y, Zeng L, Luo R, Xie K (2017). Loss of KLF4 and consequential downregulation of Smad7 exacerbate oncogenic TGF-β signaling in and promote progression of hepatocellular carcinoma. Oncogene.

[89] Tang J, Gifford CC, Samarakoon R, Higgins PJ (2018) Deregulation of negative controls on TGF-β1 signaling in tumor progression. Cancers (Basel) 6 10.3390/cancers10060159 PMC6025439 29799477

[90] Tang Y, Reissig S, Glasmacher E, Regen T, Wanke F, Nikolaev A, Gerlach K, Popp V, Karram K, Fantini MC, Schattenberg JM, Galle PR, Neurath MF, Weigmann B, Kurschus FC, Hovelmeyer N, Waisman A (2019). Alternative splice forms of CYLD mediate ubiquitination of SMAD7 to prevent TGFB signaling and promote colitis. Gastroenterology.

[91] Thompson CL, Plummer SJ, Acheson LS, Tucker TC, Casey G, Li L (2009). Association of common genetic variants in SMAD7 and risk of colon cancer. Carcinogenesis.

[92] Troncone E, Monteleone G (2019) Smad7 and colorectal carcinogenesis: a double-edged sword. Cancers (Basel) 5 10.3390/cancers11050612 PMC6563107 31052449

[93] Vishal M, Vimalraj S, Ajeetha R, Gokulnath M, Keerthana R, He Z, Partridge NC, Selvamurugan N (2017). MicroRNA-590-5p stabilizes Runx2 by targeting Smad7 during osteoblast differentiation. J Cell Physiol.

[94] von Gersdorff G, Susztak K, Rezvani F, Bitzer M, Liang D, Bottinger EP (2000). Smad3 and Smad4 mediate transcriptional activation of the human Smad7 promoter by transforming growth factor β. J Biol Chem.

[95] Wang W, Huang XR, Li AG, Liu F, Li JH, Truong LD, Wang XJ, Lan HY (2005). Signaling mechanism of TGF-β1 in prevention of renal inflammation: role of Smad7. J Am Soc Nephrol.

[96] White JR, Phillips F, Monaghan T, Fateen W, Samuel S, Ghosh S, Moran GW (2018). Review article: novel oral-targeted therapies in inflammatory bowel disease. Aliment Pharmacol Ther.

[97] Wiesner S, Ogunjimi AA, Wang HR, Rotin D, Sicheri F, Wrana JL, Forman-Kay JD (2007). Autoinhibition of the HECT-type ubiquitin ligase Smurf2 through its C2 domain. Cell.

[98] Yan X, Chen YG (2011). Smad7: not only a regulator, but also a cross-talk mediator of TGF-β signalling. Biochem J.

[99] Yan X, Liu Z, Chen Y (2009). Regulation of TGF-β signaling by Smad7. Acta Biochim Biophys Sin Shanghai.

[100] Yang F, Zhai Z, Luo X, Luo G, Zhuang L, Zhang Y, Li Y, Sun E, He Y (2020). Bioinformatics identification of key candidate genes and pathways associated with systemic lupus erythematosus. Clin Rheumatol.

[101] Yu J, Lei R, Zhuang X, Li X, Li G, Lev S, Segura MF, Zhang X, Hu G (2016) MicroRNA-182 targets SMAD7 to potentiate TGFbeta-induced epithelial-mesenchymal transition and metastasis of cancer cells. Nat Commun 13884 10.1038/ncomms13884 PMC5187443 27996004

[102] Zhang L, Huang H, Zhou F, Schimmel J, Pardo CG, Zhang T, Barakat TS, Sheppard KA, Mickanin C, Porter JA, Vertegaal AC, van Dam H, Gribnau J, Lu CX, ten Dijke P (2012). RNF12 controls embryonic stem cell fate and morphogenesis in zebrafish embryos by targeting Smad7 for degradation. Mol Cell.

[103] Zhang Z, Fan Y, Xie F, Zhou H, Jin K, Shao L, Shi W, Fang P, Yang B, van Dam H, Ten Dijke P, Zheng X, Yan X, Jia J, Zheng M, Jin J, Ding C, Ye S, Zhou F, Zhang L (2017). Breast cancer metastasis suppressor OTUD1 deubiquitinates SMAD7. Nat Commun.

[104] Zhao Y, Thornton AM, Kinney MC, Ma CA, Spinner JJ, Fuss IJ, Shevach EM, Jain A (2011). The deubiquitinase CYLD targets Smad7 protein to regulate transforming growth factor beta (TGF-β) signaling and the development of regulatory T cells. J Biol Chem.

[105] Zhou X, Zang X, Ponnusamy M, Masucci MV, Tolbert E, Gong R, Zhao TC, Liu N, Bayliss G, Dworkin LD, Zhuang S (2016). Enhancer of Zeste homolog 2 inhibition attenuates renal fibrosis by maintaining Smad7 and phosphatase and tensin homolog expression. J Am Soc Nephrol.

[106] Zhou G, Sun X, Qin Q, Lv J, Cai Y, Wang M, Mu R, Lan HY, Wang QW (2018) Loss of Smad7 promotes inflammation in rheumatoid arthritis. Front Immunol 2537 10.3389/fimmu.2018.02537 PMC6224447 30450102

[107] Zhu Z, Xu Y, Zhao J, Liu Q, Feng W, Fan J, Wang P (2015). miR-367 promotes epithelial-to-mesenchymal transition and invasion of pancreatic ductal adenocarcinoma cells by targeting the Smad7-TGF-β signalling pathway. Br J Cancer.

[108] Zulehner G, Mikula M, Schneller D, van Zijl F, Huber H, Sieghart W, Grasl-Kraupp B, Waldhor T, Peck-Radosavljevic M, Beug H, Mikulits W (2010). Nuclear β-catenin induces an early liver progenitor phenotype in hepatocellular carcinoma and promotes tumor recurrence. Am J Pathol.

